# ACE Inhibition with Captopril Retards the Development of Signs of Neurodegeneration in an Animal Model of Alzheimer’s Disease

**DOI:** 10.3390/ijms140816917

**Published:** 2013-08-16

**Authors:** Said AbdAlla, Andreas Langer, Xuebin Fu, Ursula Quitterer

**Affiliations:** 1 Molecular Pharmacology Unit, Department of Chemistry and Applied Biosciences, Swiss Federal Institute of Technology (ETH) Zurich, Zurich CH-8057, Switzerland; E-Mails: said.abdalla@pharma.ethz.ch (S.A.); andreas.langer@pharma.ethz.ch (A.L.); xuebin.fu@pharma.ethz.ch (X.F.); 2 Institute of Pharmacology and Toxicology, Department of Medicine, University of Zurich, Zurich CH-8057, Switzerland

**Keywords:** Alzheimer’s disease, amyloid precursor protein, angiotensin-converting enzyme, AT1 receptor, captopril, neurodegeneration, Tg2576 mouse model

## Abstract

Increased generation of reactive oxygen species (ROS) is a significant pathological feature in the brains of patients with Alzheimer’s disease (AD). Experimental evidence indicates that inhibition of brain ROS could be beneficial in slowing the neurodegenerative process triggered by amyloid-beta (Abeta) aggregates. The angiotensin II AT1 receptor is a significant source of brain ROS, and AD patients have an increased brain angiotensin-converting enzyme (ACE) level, which could account for an excessive angiotensin-dependent AT1-induced ROS generation. Therefore, we analyzed the impact of ACE inhibition on signs of neurodegeneration of aged Tg2576 mice as a transgenic animal model of AD. Whole genome microarray gene expression profiling and biochemical analyses demonstrated that the centrally active ACE inhibitor captopril normalized the excessive hippocampal ACE activity of AD mice. Concomitantly, the development of signs of neurodegeneration was retarded by six months of captopril treatment. The neuroprotective profile triggered by captopril was accompanied by reduced amyloidogenic processing of the amyloid precursor protein (APP), and decreased hippocampal ROS, which is known to enhance Abeta generation by increased activation of beta- and gamma-secretases. Taken together, our data present strong evidence that ACE inhibition with a widely used cardiovascular drug could interfere with Abeta-dependent neurodegeneration.

## 1. Introduction

Alzheimer’s disease (AD) is the most common form of dementia in the elderly population. The number of patients affected by AD worldwide is expected to grow substantially in the coming years due to increasing life expectancy [1]. However, available treatment options for AD are limited and ineffective to modify disease progression [2]. Research efforts of drug development aim to target major pathological features of AD, *i.e.*, the deposition of extracellular senile plaques of aggregated amyloid-beta (Aβ), and the formation of neurofibrillary tangles of aggregated hyperphosphorylated tau protein [2]. Neuropathological hallmarks of AD are considered to contribute synergistically to the process of cognitive decline and neurodegeneration [3].

Increased generation of reactive oxygen species (ROS) in the brain of AD patients is a direct consequence of Aβ-induced toxicity, which is triggered by the amyloidogenic processing of the amyloid precursor protein (APP) by sequential β-secretase and γ-secretase cleavage [4,5]. Increased oxidative stress promotes oxidative damage to proteins, lipids, DNA and RNA, all of which seem to contribute to neuronal dysfunction culminating finally in neuronal damage and cognitive impairment [6]. While Aβ contributes to the generation of ROS in AD brain, the increased oxidative stress could trigger a vicious cycle of ROS-dependent enhancement of APP cleavage, Aβ oligomerization and memory loss [7,8]. From those data, treatment strategies that combat enhanced ROS generation in mitochondria and cytosol appear as a promising approach in AD treatment [9–12].

By stimulation of the AT1 receptor, angiotensin II is a major contributor to cellular ROS generation [13–15]. Angiotensin II-dependent ROS generation is also active in neurons and brain, and plays a fundamental role in angiotensin II-dependent actions in the central nervous system [16–18]. In addition to the AT1 receptor, angiotensin II also activates the AT2 receptor, which could exert neuroprotection [19]. However, neuroprotective AT2 receptor activation appears to be compromised in the brain of AD patients by AD-related AT2 protein aggregation resulting in dysfunctional AT2 protein oligomers, which could enhance neurodegeneration [20,21].

The brain has a fully functional angiotensin system that mediates local generation of angiotensin II by the angiotensin-converting enzyme, ACE [22]. The ACE gene is a candidate gene, which could influence AD risk [23]. In addition, some studies found that AD patients have a higher frequency of the ACE gene D/D genotype, and the D-allele is associated with an increased serum ACE level, and predisposes to amnestic mild cognitive impairment [24,25]. In agreement with a putative role of ACE in AD pathogenesis, ACE protein level and activity were found increased in the brain of AD patients [26–28].

Despite the association of ACE with AD pathology, the role of ACE in AD is not clear because experimental data had shown that ACE is capable of promoting the degradation of Aβ [29]. These findings have raised concern about the use of ACE inhibitors in AD patients [29]. However, recent data from ACE deficient mice and some experimental AD models do not support the notion that ACE exerts a major role in the turnover of Aβ *in vivo* [30–32]. Likewise, ACE inhibition did not increase Aβ plaque load in some animal models of AD [31,32]. Together, these data argue against a detrimental role of ACE inhibition in AD by promoting Aβ plaque formation. In agreement with these findings, epidemiological data from patient studies showed that ACE inhibitors as a class do not increase the incidence of AD [33]. Moreover, some selected ACE inhibitors could even be beneficial by reducing the risk of dementia and AD progression [34,35]. Those AD protective effects of ACE inhibitors led to the hypothesis that brain-penetrating ACE inhibitors could exert protection against cognitive decline independent of blood pressure lowering [34,35].

Although experimental evidence also indicated beneficial effects of a brain-penetrating ACE inhibitor on prevention of cognitive decline [36], the effect of central ACE inhibition on brain Aβ deposition is not clear. Therefore, we investigated the impact of ACE inhibition on Aβ-related pathology and neurodegeneration. We chose Tg2576 mice, which express the amyloid precursor protein variant APP695 with the Swedish double mutation under control of the neuron-specific prion protein promoter [37]. These mice develop substantial Aβ plaque load and Aβ plaque-associated neuronal degeneration starting at an age of 12 months [20,37]. To investigate the effect of ACE inhibition on Aβ plaque-related pathology, we treated 12 month-old Tg2576 mice for six months with the centrally active ACE inhibitor captopril. The effect of captopril treatment on Aβ plaque development and signs of neurodegeneration was monitored by immunohistological analyses. Concomitantly, gene expression changes in the hippocampus were determined by whole genome microarray gene expression profiling. Results of our study revealed that ACE inhibition with captopril had the capacity to retard the development of Aβ plaques and Aβ-related signs of neurodegeneration.

## 2. Results and Discussion

### 2.1. ACE Inhibition with Captopril Slowed Aβ Plaque Accumulation of AD Mice

**Figure 1:**
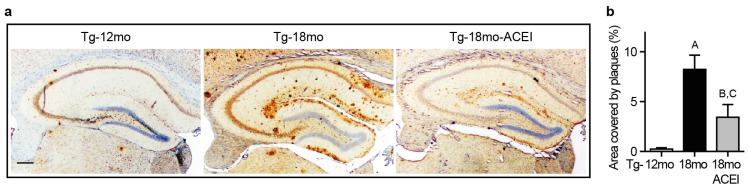
Angiotensin-converting enzyme (ACE) inhibition with captopril slowed Aβ plaque accumulation of Alzheimer’s disease (AD) mice. (**a**) Immunohistological analysis of Aβ plaque load in the hippocampus of a 12 month-old untreated Tg2576 mouse (Tg-12mo), an untreated 18 month-old Tg2576 mouse (Tg-18mo), and an 18 month-old Tg2576 mouse treated for six months with the centrally active ACE inhibitor captopril (Tg-18mo-ACEI); bar: 200 μm; (**b**) Quantitative assessment of hippocampal Aβ plaque area (±s.d., *n* = 4 mice/group; ^A,B^*p* < 0.001 *vs.* Tg-12mo; ^C^*p* < 0.01 *vs.* Tg-18mo; ANOVA and Tukey’s Multiple Comparison Test).

The impact of ACE inhibition on Aβ plaque load was determined with Tg2576 mice as AD model. Aβ plaque load was detected in the hippocampus by immunohistology with Aβ-specific antibodies on brain sections from 18 month-old Tg2576 mice treated with or without the centrally active captopril for six months (Figure 1). Brain sections representing the mean Aβ plaque load of each group show that a captopril-treated Tg2576 mouse had a substantially smaller area of hippocampal Aβ plaques compared to the untreated age-matched Tg2576 control (Figure 1a). Quantitative assessment of the antibody-stained area revealed a 58.4% ± 15.6% reduced Aβ plaque load in the hippocampus of captopril-treated Tg2576 mice compared to age-matched controls (Figure 1b). For comparison, 12 month-old Tg2576 mice were almost devoid of Aβ plaques, at the age when captopril treatment was initiated (Figure 1). These findings indicate that ACE inhibition with captopril could slow the aging-dependent accumulation of Aβ plaques in brain of Tg2576 mice.

### 2.2. Whole Genome Microarray Gene Expression Profiling of Hippocampal Genes of AD Mice upon ACE Inhibition with Captopril

**Figure 2:**
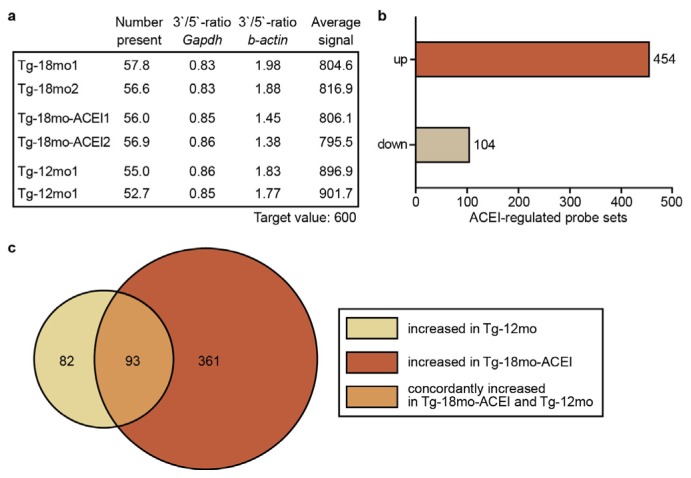
Whole genome microarray gene expression profiling of hippocampal genes of AD mice. (**a**) Hippocampal whole genome microarray gene expression profiling of 18 and 12 month-old Tg2576 (Tg-18mo and Tg-12mo) mice without or with ACE inhibitor (ACEI) treatment by captopril for six months. Hippocampal RNA from four mice was pooled for one gene chip, and two gene chips are presented for each group; (**b**) Number of significantly different probe sets of 18 month-old captopril-treated Tg2576 mice compared to untreated 18 month-old Tg2576 mice with *p* ≤ 0.01, and ≥+2-fold (up) or ≤ −2-fold (down) difference; (**c**) The Venn diagram illustrates that more than 53% (*i.e.*, 93 probe sets) of significantly increased hippocampal genes of 12 month-old Tg2576 mice compared to 18 month-old Tg2576 mice, showed concordant up-regulation in ACEI-treated 18 month-old Tg2576 mice.

We determined hippocampal gene expression changes of Tg2576 mice, which could account for the captopril-mediated slowing of Aβ plaque accumulation. Hippocampal RNA was isolated, processed for microarray gene expression profiling and hybridized to whole genome microarrays (Affymetrix GeneChip Mouse Genome MG430 2.0 Array) with more than 45,000 probe sets [38,39]. Scanning revealed uniform quality of gene chips as documented by a similar number of probe sets present, comparable 3′/5′ ratio of house-keeping genes such as *Gapdh*, and average signal intensity after normalization to a target value of 600 (Figure 2a).

We searched for significantly different probe sets between 18 month-old captopril-treated and age-matched untreated Tg2576 mice. Stringent data filtering identified 454 probe sets with significantly higher signal intensity in captopril-treated compared to untreated Tg2576 mice (≥+2-fold difference; *p* ≤ 0.01) while the signal intensity of 104 probe sets was lower of captopril-treated mice (≤ −2-fold difference; *p* ≤ 0.01) compared to the age-matched 18-month-old Tg2576 control group (Figure 2b, Tables S1 and S2). Thus, whole genome microarray gene expression profiling demonstrated that captopril treatment for six months had led to a significant modification of hippocampal gene expression.

Next, we analyzed whether captopril was capable to modify disease-related gene expression in AD mice. To determine genes involved in AD pathology, we focused on probe sets that were decreased during the accumulation of Aβ plaques, because those genes could reflect AD-related neuronal degeneration and concomitant loss of neuronal processes [20,21]. To identify those genes that were down-regulated during Aβ plaque accumulation, we compared the gene expression profile of 18 month-old Tg2576 mice with high Aβ plaque load with the gene expression profile of 12 month-old Tg2576 mice with low Aβ plaque load (cf. Figure 1). A Venn diagram shows that Aβ plaque accumulation in the hippocampus of 18 month-old Tg2576 mice was accompanied by the down-regulation of 175 probe sets relative to 12 month-old Tg2576 mice because those probe sets had a significantly higher signal intensity in 12 month-old mice (Figure 2c and Table S3). The Venn diagram also illustrates that more than 53% of those hippocampal genes (*i.e.*, 93 probe sets), were significantly increased in ACEI-treated 18 month-old Tg2576 mice, towards the pre-treatment level of 12 month-old mice (Figure 2c and Table S4). That observation indicates that captopril could preserve the expression level of a substantial number of genes, which were down-regulated during Aβ plaque accumulation of AD mice.

### 2.3. Captopril Prevented the Down-Regulation of Genes Involved in Neuronal Regeneration and Cognition

Gene ontology (GO) analysis was used to categorize those genes that were preserved by captopril treatment, but down-regulated during Aβ plaque accumulation of Tg2576 mice.

**Figure 3:**
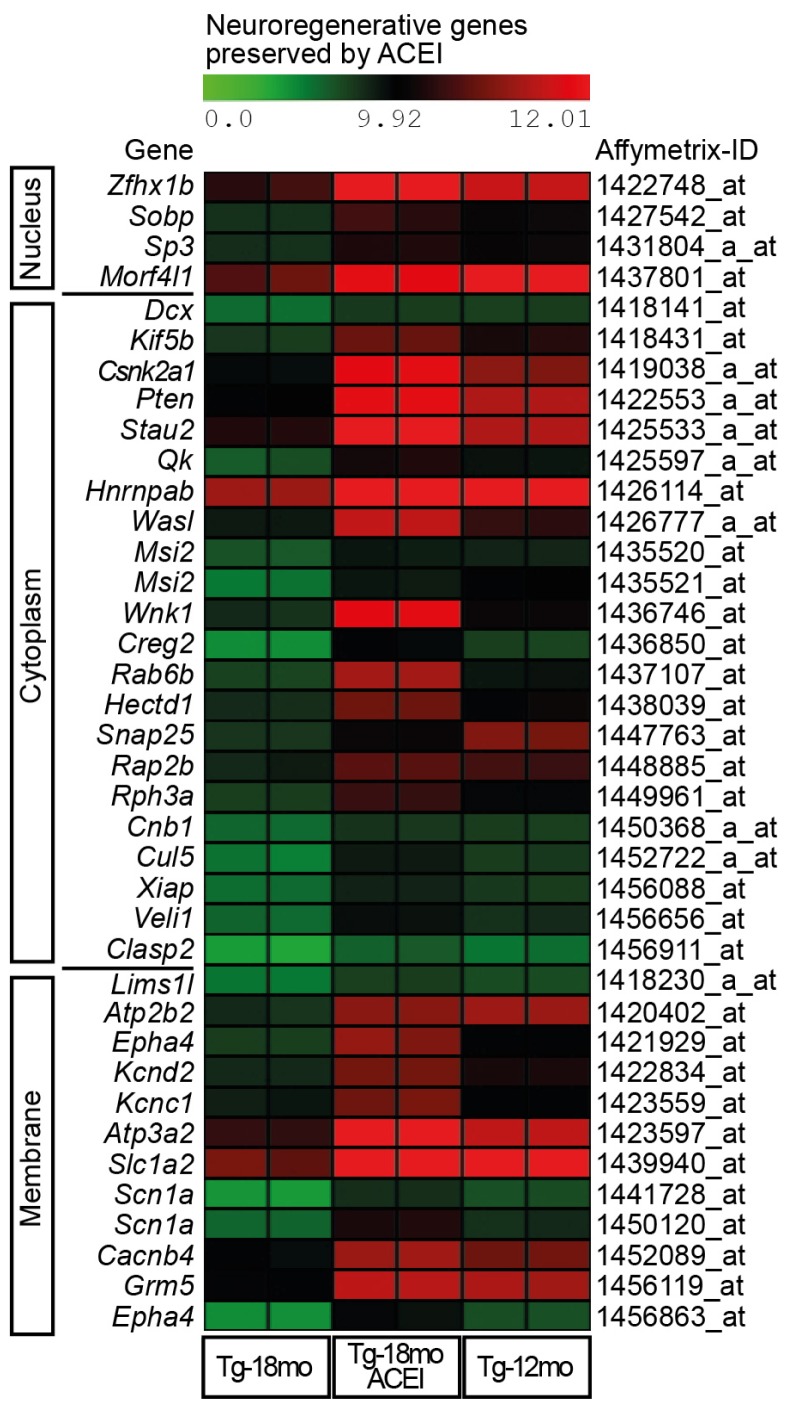
Captopril prevented the AD-related down-regulation of hippocampal genes involved in neuronal regeneration and cognition. A heat map visualizes signal intensities (log2-transformed data, centered to the median value) of significantly different probe sets of hippocampal genes involved in neuronal regeneration and cognition, which were down-regulated during Aβ plaque accumulation of 18 month-old Tg2576 mice and preserved by treatment with the ACE inhibitor (ACEI) captopril. Probe sets of 12 month-old Tg2576 mice are also shown to illustrate pre-treatment condition. Analysis of variance with *p*-values is presented as Table S5.

GO analysis identified that 38 AD-related probe sets that were preserved by captopril, had established functions in neuronal regeneration and cognition. Identified gene products belonged to all major cellular components, *i.e.*, nucleus, cytoplasm, and plasma membrane (Figure 3 and Table S5). A heat map visualizes signal intensities of identified probe sets to illustrate the preservation of neuronal gene expression by captopril (Figure 3 and Table S5).

### 2.4. ACE Inhibition Promoted Signs of Neuronal Regeneration and Up-Regulated the Expression of Genes Involved in the Formation of Neuronal Membrane and Neuronal Processes

**Figure 4:**
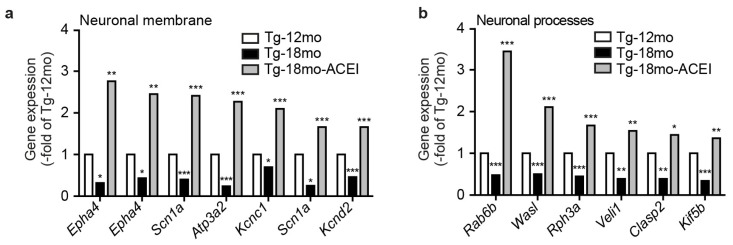

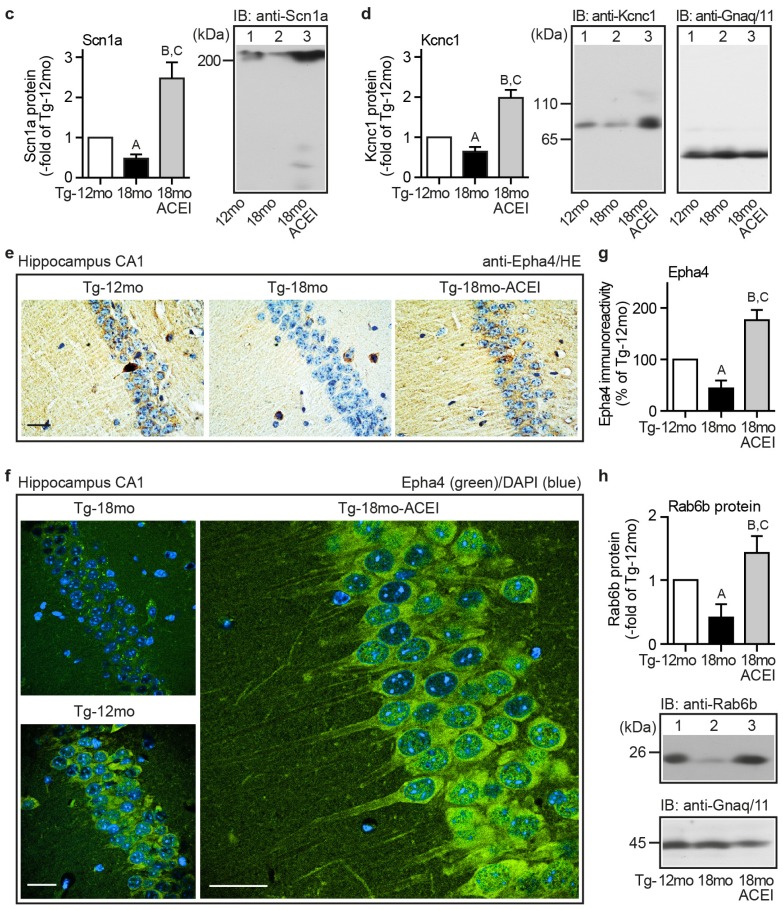
ACE inhibition promoted signs of neuronal regeneration and up-regulated the expression of genes involved in the formation of neuronal membrane and neuronal processes. (**a,b**) Microarray gene expression data of hippocampal genes categorized by gene ontology (GO) terms “neuronal membrane” (**a**), and “neuronal processes” (**b**), which were up-regulated by captopril treatment over the expression level of untreated 12 month-old Tg2576 controls (***p < 0.001, **p < 0.01 and *p < 0.05 *vs.* Tg-12mo; ANOVA and Tukey’s Multiple Comparison Test, no false discovery correction; the average signal intensity from two gene chips is presented as -fold of Tg-12mo, with RNA pooled from four mice for one gene chip). The following probe sets were identified: 1456863_at (*Epha4*), 1421929_at (*Epha4*), 1450120_at (*Scn1a*), 1423597_at (*Atp3a2*), 1423559_at (*Kcnc1*), 1441728_at (*Scn1a*), 1422834_at (*Kcnd2*), 1437107_at (*Rab6b*), 1426777_a_at (*Wasl*), 1449961_at (*Rph3a*), 1456656_at (*Veli1*), 1456911_at (*Clasp2*), 1418431_at (*Kif5b*); (**c**,**d**) Immunoblot detection of hippocampal protein level of Scn1a (**c**) and Kcnc1 (**d**). Left panels show quantitative data evaluation (±s.d.; *n* = 4 mice/group; ^A^*p* < 0.05 (**c**), ^A^*p* < 0.01 (**d**), and ^B^*p* < 0.001 *vs.* Tg-12mo (**c**,**d**); ^C^*p* < 0.001 *vs.* Tg-18mo (**c**,**d**); ANOVA and Tukey’s Multiple Comparison Test). Middle/right panels show a representative immunoblot experiment; (**e**) Immunohistological detection of Epha4 in hippocampal CA1 neurons of an 18 month-old captopril-treated (ACEI) Tg2576 mouse compared to untreated 18 month-old (Tg-18mo) and 12 month-old (Tg-12mo) Tg2576 mice. Nuclei were stained with hematoxylin (HE), bar 25 μm; (**f**) Immunofluorescence detection of Epha4 with Epha4-specific antibodies (green) in hippocampal CA1 neurons of an 18 month-old captopril-treated (ACEI) Tg2576 mouse compared to untreated 18 month-old and 12 month-old Tg2576 (Tg) mice. Nuclei were stained with DAPI (blue), bar 25 μm. Immunohistological (**e**) and immunofluorescence (**f**) experiments are representative of four mice/group; (**g**) Hippocampal Epha4 content from different groups of Tg2576 mice was quantified by direct binding assay with anti-Epha4 antibodies followed by detection with ^125^I-labeled secondary antibodies (±s.d.; *n* = 4 mice/group; ^A^*p* < 0.01 and ^B^*p* < 0.001 *vs.* Tg-12mo; ^C^*p* < 0.001 *vs.* Tg-18mo; ANOVA and Tukey’s Multiple Comparison Test); (**h**) Immunoblot detection of hippocampal Rab6b protein of the different treatment groups. The upper panel shows quantitative data evaluation (±s.d.; *n* = 4 mice/group; ^A^*p* < 0.01 and ^B^*p* < 0.05 *vs.* Tg-12mo; ^C^*p* < 0.001 *vs.* Tg-18mo; ANOVA and Tukey’s Multiple Comparison Test), and middle and lower panels show a representative immunoblot experiment.

Captopril prevented the Aβ-induced down-regulation of neuronal genes. We next asked whether captopril “actively” up-regulated hippocampal gene expression over the expression level of the 12 month-old Tg2576 control group. Microarray data filtering identified eleven hippocampal genes detected by 13 different probes, which were significantly up-regulated by captopril and categorized by GO terms “neuronal membrane” and “neuronal processes” (Figure 4a,b).

Among up-regulated gene products involved in formation of the “neuronal membrane”, a literature search identified several genes, which were previously shown to be reduced in AD animal models or AD patients, *i.e.*, *Epha4* [40], *Scn1a* [41], and *Kcnc1* [42]. Protein detection by immunoblot was used to validate some of the microarray gene expression data with different cohorts of mice. Immunoblotting showed that captopril had prevented the AD-related down-regulation of hippocampal Scn1a, *i.e.*, the sodium channel, voltage gated, type I, alpha (Figure 4c). Moreover, captopril had promoted a significant increase in hippocampal Scn1a protein content over the level of 12 month-old Tg2576 mice (Figure 4c). Similar results were obtained with Kcnc1, *i.e*., the potassium voltage gated channel, Shaw-related subfamily, member 1 (Figure 4d).

To further validate microarray data, we focused on Epha4, the Ephrin type-A receptor 4, which is reduced in the hippocampus of AD mice at an early stage, before the onset of overt cognitive impairment [40]. Epha4 is also reduced in hippocampal tissue from AD patients [40]. Immunohistological analysis confirmed the microarray data for Epha4 and showed a high protein level of Epha4 in hippocampal CA1 neurons of an 18 month-old captopril-treated Tg2576 mouse compared to untreated 12 month-old and 18 month-old Tg2576 mice (Figure 4e). Similar results were obtained by immunofluorescence detection of Epha4 (Figure 4f). Moreover, immunofluorescence analysis revealed that captopril treatment had promoted the regeneration of Epha4-positive CA1 neuronal processes, which showed degeneration in 18 and 12 month-old Tg2576 mice (Figure 4f, left panels *vs.* right panel). Those findings complement previous data, which linked the AD-related down-regulation of Epha4 to neuronal degeneration and synapse loss [40]. Quantitative assessment of hippocampal Epha4 protein further confirmed that captopril had up-regulated the Epha4 protein content over the level of untreated 12 month-old Tg2576 mice (Figure 4g).

In agreement with the capacity of captopril to promote signs of neuronal regeneration, captopril treatment also led to an increased hippocampal Rab6b level, a protein expressed in neuronal processes, while Rab6b was decreased in untreated Tg2576 mice with increasing age (Figure 4h). Taken together, our data strongly suggest that captopril treatment retarded the AD-related decline of neuronal gene expression and triggered signs of neuronal regeneration.

### 2.5. Captopril Treatment Prevented the AD-Related Up-Regulation of ACE and Reduced the Hippocampal Angiotensin II Level of AD Mice

**Figure 5:**
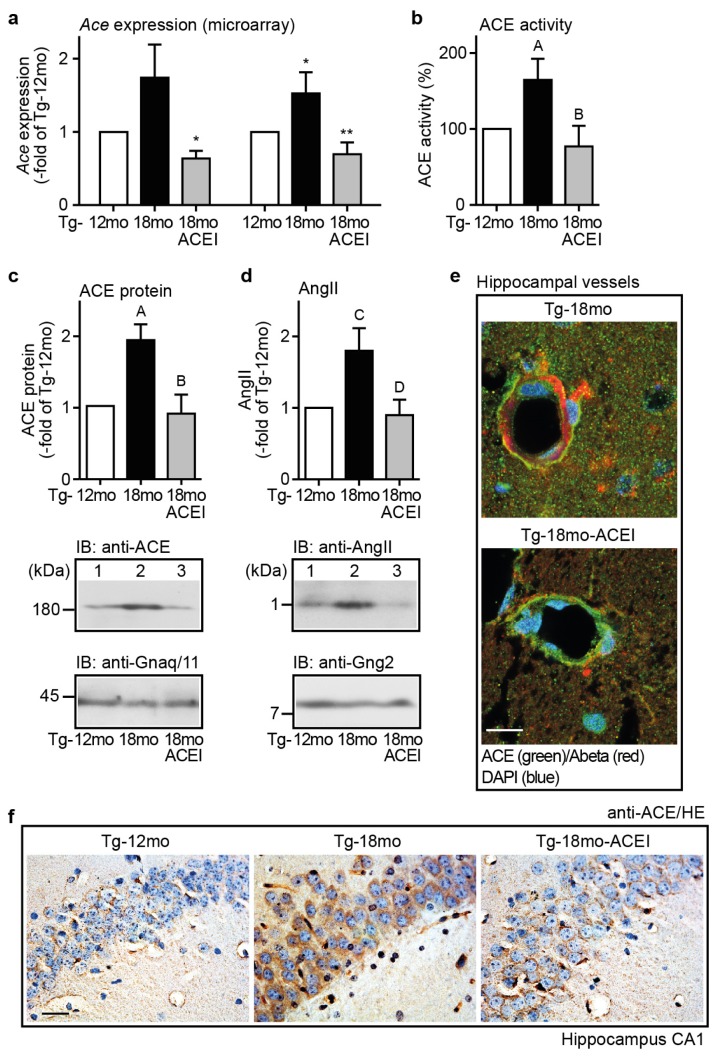
Captopril prevented the up-regulation of ACE and reduced the hippocampal angiotensin II level of AD mice. (**a**) Microarray gene expression data of *ACE* gene expression (±s.d.; *n* = 2 gene chips/group, with RNA from four mice pooled for one gene chip; *p < 0.05 for Affymetrix ID 1427034_at (left), and **p < 0.01 for Affymetrix ID 1451911_a_at (right), *vs.* Tg-18mo; *p < 0.05 for Affymetrix ID 1451911_a_at, *vs.* Tg-12mo; ANOVA and Tukey’s Multiple Comparison Test); (**b**) Hippocampal ACE activity of 18 month-old Tg2576 (Tg) mice was normalized by captopril (ACEI) treatment (±s.d.; *n* = 4 mice/group; ^A^*p* < 0.01 *vs.* Tg-12mo; ^B^*p* < 0.001 *vs.* Tg-18mo; ANOVA and Tukey’s Multiple Comparison Test); (**c**,**d**) Quantitative immunoblot analysis of ACE protein level (**c**), and angiotensin II peptide level (**d**) of different groups of AD mice (±s.d.; *n* = 4 mice/group; ^A^*p* < 0.001 *vs.* Tg-12mo, ^B^*p* < 0.001 *vs.* Tg-18mo (**c**), and ^C^*p* < 0.01 *vs.* Tg-12mo, ^D^*p* < 0.001 *vs.* Tg-18mo (**d**); ANOVA and Tukey’s Multiple Comparison Test); (**e**) Immunofluorescence analysis with anti-ACE and anti-Aβ antibodies revealed vascular localization of ACE (green) and Aβ (red) in hippocampal vessels of untreated and treated 18 month-old Tg2576 mice. Nuclei were stained with DAPI (blue; bar 25 μm); (**f**) Immunohistological detection of ACE in hippocampal CA1 neurons of 12 and 18 month-old Tg2576 (Tg) mice treated without or with captopril (ACEI). Nuclei were stained with hematoxylin (HE), bar: 25 μm. Immunofluorescence (**e**) and immunohistological (**f**) data are representative of four mice/group.

In view of the neuro-regenerative potential of the ACE inhibitor captopril, we investigated the hippocampal expression of ACE in AD mice because the ACE level is also significantly increased in the brains of AD patients and correlates directly with disease stage and Aβ load [28,43]. Microarray gene expression data showed that the aging-dependent increase in Aβ plaque load of Tg2576 mice was accompanied by a substantial rise in hippocampal ACE gene expression (Figure 5a). That increase in ACE gene expression was completely prevented by captopril (Figure 5a).

Concomitantly, ACE activity measurement showed that captopril-treated mice had a significantly lower hippocampal ACE activity than age-matched untreated Tg2576 mice (Figure 5b). Immunoblot analysis with ACE-specific antibodies confirmed gene expression data and showed that hippocampal ACE protein level was normalized by captopril (Figure 5c). In agreement with ACE inhibition, captopril treatment was accompanied by a significantly reduced hippocampal angiotensin II level compared to untreated 18 month-old AD mice (Figure 5d). Taken together, captopril treatment had prevented the Aβ-related increase in hippocampal ACE protein and ACE activity of AD mice.

### 2.6. ACE Protein in Hippocampal Vessels and Neurons of AD Mice Could Account for a Hippocampal Action of Captopril

Previous data indicated that ACE was localized in the perivascular region of brain cortical vessels of AD patients [27,28]. To localize the ACE protein in brain of Tg2576 mice, we used immunofluorescence microscopy. Immunofluorescence analysis with ACE-specific antibodies localized the ACE protein in hippocampal vessels of untreated and captopril-treated 18 month-old Tg2576 mice (Figure 5e). The ACE protein was localized in close proximity to vascular Aβ deposits in a representative vessel of an untreated 18 month-old Tg2576 mouse (Figure 5e, upper panel), while the vessel of a captopril-treated Tg2576 mouse showed a reduced vascular Aβ load (Figure 5e, lower panel).

In addition to vascular cells, immunohistological analysis localized the ACE protein also in cell soma of hippocampal CA1 neurons of Tg2576 mice (Figure 5f). In agreement with immunoblot data, the hippocampal ACE protein level was substantially increased in untreated 18 month-old Tg2576 mice whereas ACE immunostaining was less in the captopril-treated Tg2576 mouse (Figure 5f). Thus, Tg2576 mice resemble AD patients by showing increased ACE protein level with advancement of AD pathology [27,28,43]. Notably, the up-regulation of ACE could be a direct consequence of increased Aβ plaque load [28,43,44]. Treatment with captopril prevented the Aβ-related ACE protein up-regulation and reduced the angiotensin II generation in the hippocampus of AD mice.

**Figure 6:**
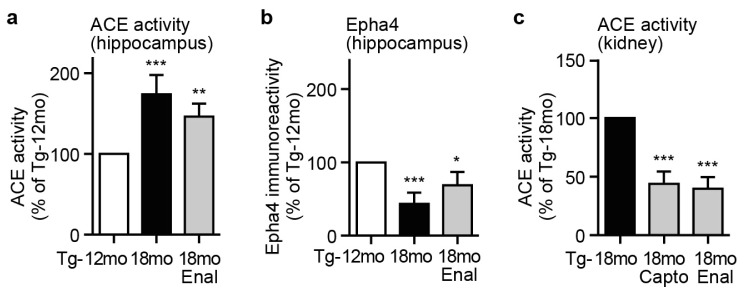
Treatment of AD mice with a predominantly peripheral-acting ACE inhibitor. (**a**) Hippocampal ACE activity of 12 and 18 month-old Tg2576 mice treated without or with enalapril (Enal) for six months (±s.d.; *n* = 4 mice/group; ***p < 0.001 and ^**^p < 0.01 *vs.* Tg-12mo; ANOVA and Tukey’s Multiple Comparison Test); (**b**) Hippocampal Epha4 content of Tg2576 mice treated without or with enalapril (Enal) was determined by direct binding assay with anti-Epha4 antibodies followed by detection with ^125^I-labeled secondary antibodies (±s.d.; *n* = 4 mice/group; ***p < 0.001 and *p < 0.05 *vs.* Tg-12mo; ANOVA and Tukey’s Multiple Comparison Test); (**c**) Comparable peripheral ACE inhibition by enalapril and captopril was shown by renal ACE activity measurement of Tg2576 mice treated for six months with captopril (Capto) or enalapril (Enal) as indicated (±s.d.; *n* = 4 mice/group; ***p < 0.001 *vs.* Tg-18mo; ANOVA and Tukey’s Multiple Comparison Test).

That effect could be related to a central action of captopril because treatment of Tg2576 mice with a predominantly peripheral-acting ACE inhibitor, enalapril (enalaprilat), for six months did not significantly alter the hippocampal ACE activity of 18 month-old Tg2576 mice (Figure 6a). In addition, enalapril did not prevent the down-regulation of hippocampal Epha4, an indicator of neuronal degeneration (Figure 6b). As a control, peripheral actions of enalapril and captopril were comparable, as documented by the inhibition of renal ACE activity (Figure 6c).

### 2.7. Co-Localization of the AT1 Receptor with Aβ in Hippocampal Neurons of AD Mice

**Figure 7:**
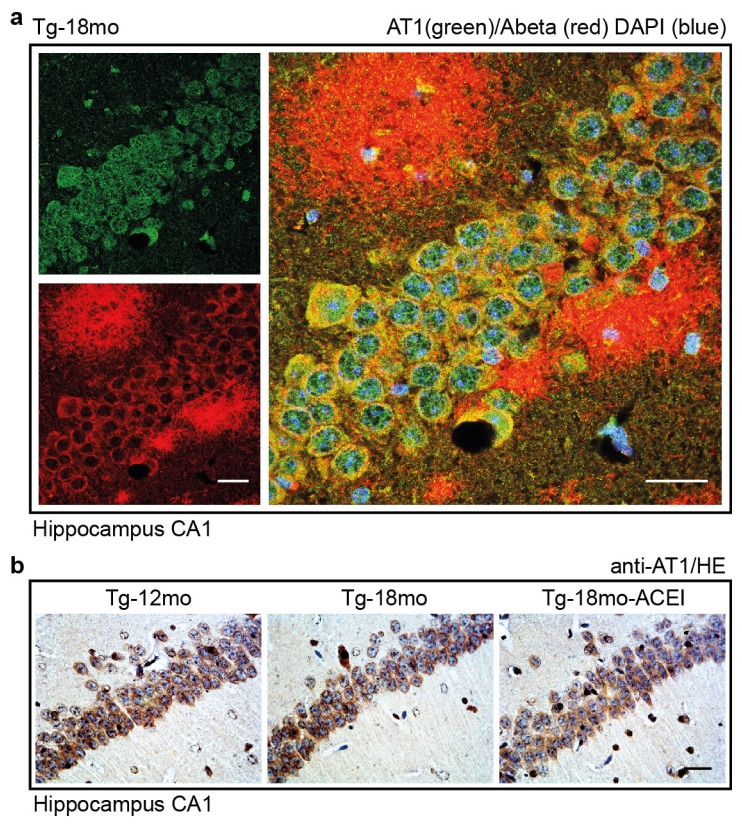

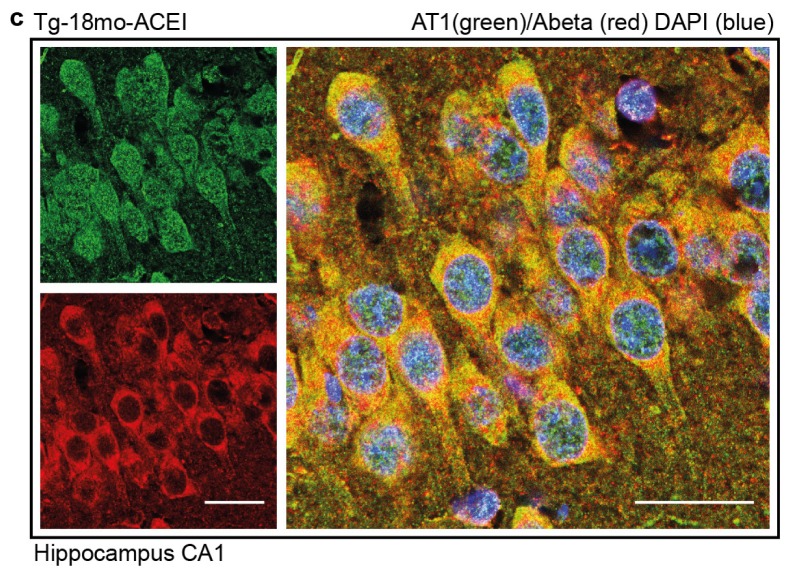
Co-localization of the AT1 receptor with Aβ in hippocampal neurons of AD mice. (**a**) Immunofluorescence localization of AT1 (green) and Aβ (red) in hippocampal neurons of an untreated 18 month-old Tg2576 (Tg) mouse. Nuclei were stained with DAPI (blue), bar: 25 μm; (**b**) Immunohistological detection of AT1 in the hippocampus of 12 month-old, and 18 month-old Tg2576 (Tg) mice treated without or with the ACE inhibitor (ACEI) captopril as indicated. Nuclei were stained with hematoxylin, HE (bar: 25 μm); (**c**) Immunofluorescence analysis revealed cytosolic Aβ of AT1-positive CA1 neurons of an 18 month-old captopril-treated (ACEI) Tg2576 mouse. Intact neuronal processes of AT1-positive neurons could reflect the neuroregenerative activity of captopril. Nuclei were stained with DAPI (blue), bar 25 μm. Immunofluorescence (**a**,**c**) and immunohistological (**b**) data are representative of four mice/group.

The Aβ-related increase in ACE protein [28,43,44] could enhance the angiotensin II-mediated activation of the AT1 receptor in brain of AD mice. Since the brain AT1 receptor stimulates Aβ generation by enhancing the amyloidogenic processing of APP [45], we determined the localization of the AT1 receptor in Tg2576 mice, and asked for the potential co-localization of AT1 with Aβ in the hippocampus of AD mice (Figure 7a). We focused on the hippocampal CA1 region because of its high sensitivity to AD-related neurodegeneration [20,21]. Moreover, CA1 neurons of aged AD mice had a high level of ACE protein (cf. Figure 5f). Immunofluorescence microscopy revealed substantial AT1 immunoreactivity in hippocampal CA1 neurons of an untreated 18 month-old Tg2576 mouse (Figure 7a). The AT1-positive neurons were localized in close proximity to Aβ plaques (Figure 7a), a trigger of ACE [43,44].

In addition to 18 month-old AD mice with high Aβ plaque load, AT1 receptor-positive hippocampal CA1 neurons were also detected by immunohistological staining in mice with lower Aβ plaque level, *i.e.*, untreated 12 month-old and captopril-treated 18 month-old Tg2576 mice (Figure 7b).

We used immunofluorescence microscopy to localize AT1 in CA1 neurons of captopril-treated Tg2576 mice. Analogous to Epha4-positive CA1 neurons of captopril-treated mice (cf. Figure 4e,f), AT1-positive CA1 neurons of captopril-treated mice displayed intact neuronal processes, even in the presence of cytosolic Aβ (Figure 7c). This observation could be another indication for the neuro-protective and neuro-regenerative potential of the ACE inhibitor captopril.

Taken together, our data are compatible with the notion that the Aβ-induced increase in ACE protein of aged AD mice leads to enhanced hippocampal angiotensin II generation, which could promote AT1 receptor activation of adjacent neurons and subsequent AT1-dependent Aβ generation [45]. Vice versa, inhibition of ACE-dependent angiotensin II generation by captopril could block the activation of those hippocampal AT1 receptors.

### 2.8. Captopril Treatment Reduced Hippocampal ROS and Protein Oxidation

**Figure 8:**
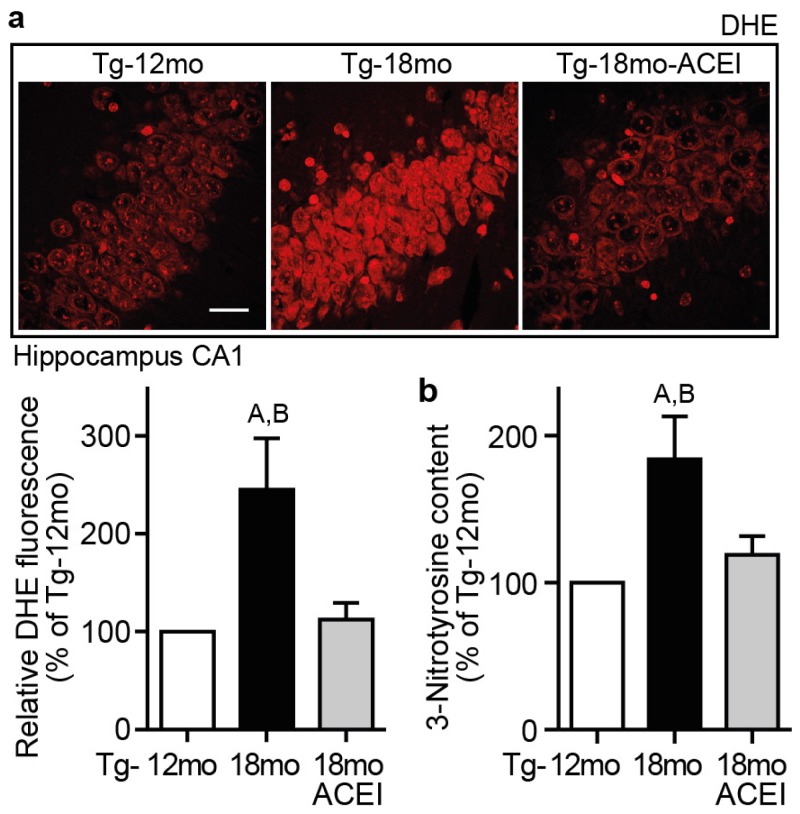
Captopril treatment reduced hippocampal ROS and protein oxidation. (**a**) Increased superoxide generation of 18 month-old Tg2576 (Tg-18mo) mice was normalized by captopril (ACEI) treatment as determined by dihydroethidium staining (DHE) of hippocampal sections, bar: 25 μm. The lower panel presents relative DHE fluorescence levels (±s.d.; *n* = 4 mice/group; ^A,B^*p* = 0.001 *vs.* Tg-12mo and Tg-18mo-ACEI; ANOVA and Tukey’s Multiple Comparison Test); (**b**) The increase in hippocampal 3-nitrotyrosine content of 18 month-old Tg2576 mice (Tg-18mo) was prevented by captopril (±s.d.; *n* = 4 mice/group; ^A^*p* < 0.001 *vs.* Tg-12mo and ^B^*p* < 0.01 *vs.* Tg-18mo-ACEI; ANOVA and Tukey’s Multiple Comparison Test).

AT1 receptor activation by angiotensin II is a substantial trigger of brain ROS [16–18], and ROS enhances the amyloidogenic processing of APP as well as Aβ plaque formation [7,8]. Therefore, we asked whether inhibition of ACE-dependent angiotensin II generation by captopril could affect the level of hippocampal ROS. We determined the amount of hippocampal superoxide content as a major ROS form by dihydroethidium (DHE) staining (Figure 8a). Dihydroethidium staining revealed an aging-dependent increase in the superoxide content of hippocampal CA1 neurons of untreated 18 month-old Tg2576 mice with high Aβ plaque load compared to 12 month-old Tg2576 mice with low Aβ plaque load (Figure 8a). This increase in superoxide generation was largely prevented by captopril treatment (Figure 8a).

In agreement with decreased superoxide generation, captopril treatment also led to a reduced protein oxidation, *i.e.*, the aging-related increase in hippocampal 3-nitrotyrosine content of 18 month-old Tg2576 mice, a marker of protein oxidation of AD brain [46], was prevented in captopril-treated Tg2576 mice (Figure 8b).

### 2.9. Captopril Treatment Reduced Markers of Amyloidogenic Processing of APP

**Figure 9:**
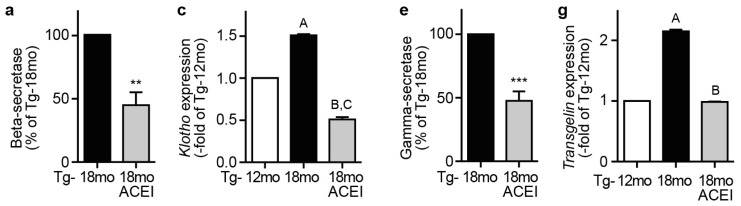

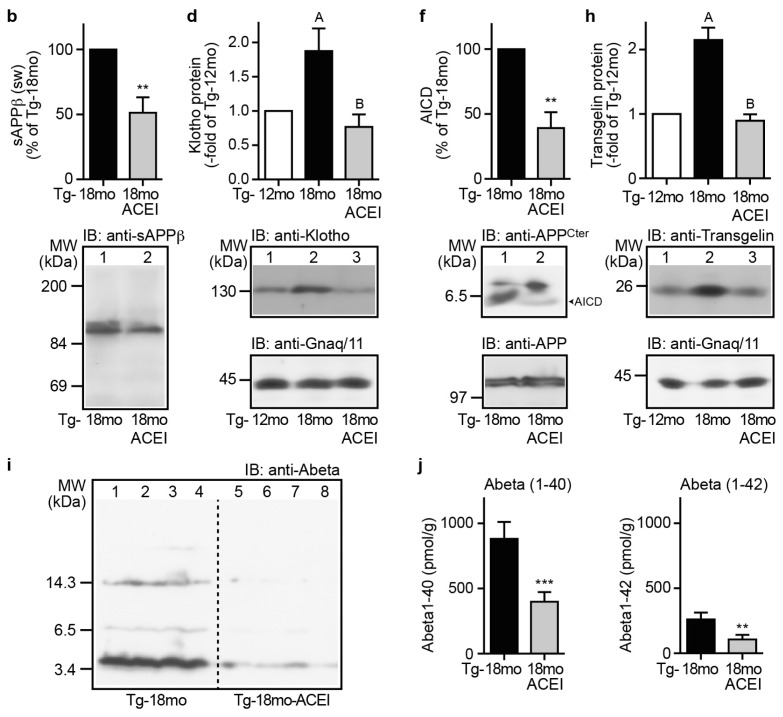
Captopril treatment reduced markers of amyloidogenic processing of APP. (**a**) Hippocampal β-secretase activity of 18 month-old Tg2576 mice treated without or with captopril (ACEI) for six months as indicated (±s.d.; *n* = 4 mice/group; **p = 0.0019); (**b**) Hippocampal content of sAPPβ (sw) of 18 month-old Tg2576 mice (Tg-18mo) treated without or with captopril (ACEI) for six months was determined by immunoblot with sAPPβ (sw)-specific antibodies (±s.d.; *n* = 4 mice/group; **p = 0.0038); (**c**) Hippocampal microarray gene expression data of *Klotho* (Affymetrix ID: 1423400_at; ±s.d.; *n* = 2 gene chips/group with RNA from four mice pooled for one gene chip; ^A,B^*p* < 0.01 *vs.* Tg-12mo; ^C^*p* < 0.001 *vs.* Tg-18mo; ANOVA and Tukey’s Multiple Comparison Test); (**d**) Determination of hippocampal Klotho protein level by quantitative immunoblotting (±s.d.; *n* = 4 mice/group; ^A^*p* < 0.001 *vs.* Tg-12mo and ^B^*p* < 0.001 *vs.* Tg-18mo; ANOVA and Tukey’s Multiple Comparison Test); (**e**) Hippocampal γ-secretase activity of 18 month-old Tg2576 mice treated without or with captopril (ACEI) for six months as indicated (±s.d.; *n* = 4 mice/group; ***p = 0.0008); (**f**) Immunoblot quantification of hippocampal AICD content of 18 month-old Tg2576 mice (Tg-18mo) treated without or with captopril (ACEI) for six months (±s.d.; *n* = 4 mice/group; **p = 0.0021). Middle and lower panels show representative immunoblot detections of AICD and APP; (**g**) Hippocampal microarray gene expression data of *Transgelin* (Affymetrix ID: 1423505_at; ±s.d.; *n* = 2 gene chips/group with RNA from four mice pooled for one gene chip; ^A^*p* < 0.05 *vs.* Tg-12mo; and ^B^*p* < 0.05 *vs.* Tg-18mo; ANOVA and Tukey’s Multiple Comparison Test); (**h**) Determination of hippocampal Transgelin protein level by quantitative immunoblotting (±s.d.; *n* = 4 mice/group; ^A^*p* < 0.001 *vs.* Tg-12mo and ^B^*p* < 0.001 *vs.* Tg-18mo; ANOVA and Tukey’s Multiple Comparison Test); (**i**) Immunoblot detection of Aβ peptides in guanidine-extracted hippocampal tissue of 18 month-old Tg2576 (Tg-18mo) mice treated without or with captopril (ACEI) for six months (*n* = 4 mice/group); (**j**) Quantitative determination of SDS-insoluble hippocampal Aβ^1-40^ and Aβ^1-42^ content of 18 month-old Tg2576 (Tg-18mo) mice treated without or with captopril (ACEI) for six months (±s.d.; *n* = 4 mice/group; ***p = 0.0006 and **p = 0.0029).

ROS promotes the amyloidogenic processing of Aβ by enhancing β-secretase- and γ-secretase-mediated APP cleavage [7,47]. In addition, brain angiotensin II infusion enhanced the activities of β- and γ-secretase by AT1 receptor stimulation [45]. In agreement with a ROS/angiotensin II-dependent effect on β-secretase activity, enzyme activity measurement showed that the captopril-mediated decrease in hippocampal ROS/angiotensin II generation was accompanied by a significant decrease in β-secretase activity, *i.e*., β-secretase activity was 55.7% ± 10.8% lower in captopril-treated mice compared to untreated 18 month-old Tg2576 mice (Figure 9a). Concomitantly, the hippocampal content of the *N*-terminal fragment of APP^Swe^ generated by β-secretase cleavage [sAPPβ (sw)] was significantly reduced of captopril-treated Tg2576 mice compared to untreated 18 month-old Tg2576 mice (Figure 9b).

We asked whether the hippocampal gene expression profile reflected the captopril-induced change in β-secretase activity of Tg2576 mice. To this end, we focused on *Klotho* because the *N*-terminal fragment of APP generated by β-secretase cleavage (sAPPβ) is reported to induce the transcription of *Klotho* [48]. In parallel to the decrease in β-secretase activity, captopril treatment led to a significantly reduced hippocampal expression and protein level of *Klotho* compared to age-matched untreated 18 month-old Tg2576 mice (Figure 9c,d). Together these findings indicate that the captopril-induced decrease in ROS/angiotensin II level was accompanied by reduced β-secretase-activity, which could account for decreased amyloidogenic processing of APP.

After APP cleavage by β-secretase, cleavage by γ-secretase finally releases Aβ. In agreement with ROS/angiotensin II-dependent enhancement of γ-secretase activity [7,45], hippocampal γ-secretase activity of captopril-treated mice was significantly reduced by 52.5% ± 7.5% compared to age-matched untreated AD mice (Figure 9e). In agreement with decreased γ-secretase activity, the hippocampal content of the APP intracellular domain (AICD), which is the *C*-terminal product of γ-secretase cleavage of APP, was significantly lower of captopril-treated 18 month-old Tg2576 mice compared to age-matched untreated AD mice (Figure 9f). Since increasing evidence suggests that AICD contributes to AD pathogenesis by promoting signs of neurodegeneration [49], that observation could indicate that the neuro-regenerative profile of captopril could be partially attributed to a decrease of AICD.

Searching for signs of γ-secretase activity in the hippocampal gene expression profile, we focused on *Transgelin* because previous data had shown that the γ-secretase-mediated *C*-terminal cleavage product of APP, *i.e.*, the APP intracellular domain (AICD), triggers the expression of *Transgelin* [50]. Concomitantly with a decrease in γ-secretase activity, the expression level of *Transgelin* was significantly lower in captopril-treated Tg2576 mice compared to age-matched untreated Tg2576 mice (Figure 9g). Immunoblot analysis confirmed the gene expression data and showed that captopril had prevented the AD-induced rise in Transgelin protein level of aged 18 month-old Tg2576 mice (Figure 9h).

Reduced amyloidogenic processing of APP upon captopril treatment was also reflected by the level of hippocampal Aβ peptides. Immunoblot detection showed a significantly lower level of Aβ peptides in hippocampal tissue extracts of captopril-treated Tg2576 mice relative to age-matched untreated controls (Figure 9i). Quantitative assessment of SDS-insoluble Aβ peptides by ELISA confirmed the decrease in hippocampal Aβ^1-40^ and Aβ^1-42^ peptides upon captopril treatment (Figure 9j).

### 2.10. Model of Vicious Cycle of Angiotensin II-Dependent Aβ Generation

**Figure 10:**
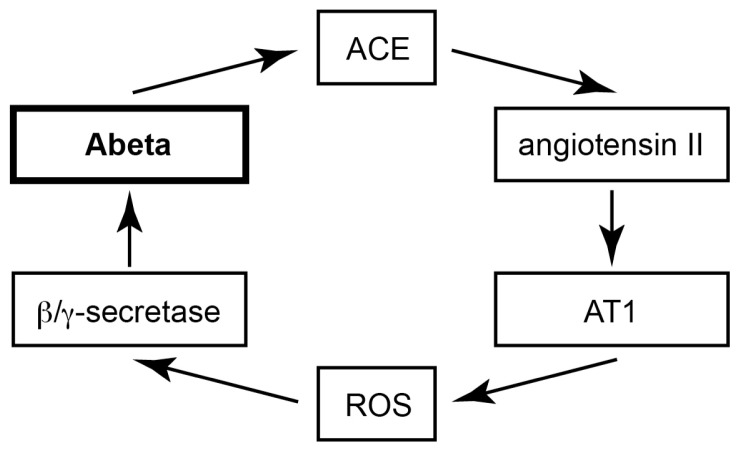
ACE seems to be part of a vicious cycle of angiotensin II-AT1-dependent Aβ generation.

Taken together our data present strong evidence that captopril retarded the aging-dependent accumulation of Aβ plaques in brain of Tg2576 mice by mediating a decrease of β- and γ-secretase-dependent amyloidogenic processing of APP. Concomitantly, treatment with the centrally active ACE inhibitor captopril could slow the development of signs of neuronal degeneration in the Tg2576 mouse model of AD. In view of previous data demonstrating that ACE is induced by aggregated Aβ *in vitro* and *in vivo* [43,44], our findings suggest a vicious cycle, which seems to contribute to disease progression in AD: (i) Augmented Aβ level of AD brain induces ACE [43,44]. (ii) The increased ACE protein generates angiotensin II. (iii) Angiotensin II stimulates AT1-dependent ROS generation and enhances amyloidogenic APP processing by β- and γ-secretase [7,45,47], thereby accelerating Aβ-mediated ACE induction and angiotensin II-AT1-dependent Aβ generation (Figure 10). Captopril treatment could interfere with that vicious cycle by normalizing ACE protein level and activity. This effect could account for decreased angiotensin II-dependent ROS generation, and reduced amyloidogenic processing of APP and Aβ generation in AD mice. While our study provided evidence for a beneficial effect of captopril in the Tg2576 mouse model, future studies are needed to determine the impact of the angiotensin II system on the Aβ-induced neurodegenerative process of other experimental AD models or AD patients.

## 3. Experimental Section

### 3.1. Animal Model of AD and Captopril Treatment

The study used male Tg2576 mice on a C57BL/6J (B6) background, which expresses the human APP695 isoform with the Swedish double mutation (APP695^K670N,M671L^; APP^Swe^) under control of the neuron-specific prion protein promoter [37]. Treatment with captopril or enalapril (20 mg/kg/day or 25 mg/kg/day in drinking water, dissolved fresh every day) was initiated at 12 months of age when Tg2576 mice start to develop substantial Aβ plaque load and signs of overt neurodegeneration [20,21,37]. ACE inhibitor treatment was well tolerated, and the applied doses are effective in mice as documented by previous studies [38,51]. Treatment of Tg2576 mice was performed for six months, from 12–18 months of age. As control groups, we used 18 month-old Tg2576 mice, which received drinking water without ACE inhibitor, and 12 month-old Tg2576 mice. Mice were randomly assigned to different treatment groups. All mice were kept on a 12 h light/12 h dark regime, had free access to food and water, and were fed a standard rodent chow. At the end of the observation period, Tg2576 mice were anesthetized with ketamine and xylazine (100 mg/kg and 10 mg/kg, i.p.), perfused intracardially with sterile PBS, brains were isolated, and hippocampi were dissected and immediately frozen in liquid nitrogen or processed for further use. Animal experiments were performed in accordance with NIH guidelines, and were reviewed and approved by the local committee on animal care and use (University of Zurich, Switzerland).

### 3.2. Whole Genome Microarray Gene Expression Profiling

Whole genome microarray gene expression profiling of hippocampal gene expression was performed similarly as described [38,39]. Briefly, total RNA was isolated from mouse hippocampi by a commercial kit (RNeasy mini kit, Qiagen GmbH, Hilden, Germany), hippocampal RNA was reverse transcribed and processed for whole genome microarray gene expression profiling according to the protocol of the manufacturer (Affymetrix GeneChip Expression Analysis Technical Manual, rev. 5, Affymetrix Inc., Santa Clara, CA, USA). Fragmented, biotin-labeled cRNA (15 μg/gene chip) was hybridized to the microarray gene chip (Affymetrix GeneChip Mouse Genome MG430 2.0 Array with more than 45,000 probe sets), in 200 μL of hybridization solution in a Hybridization Oven 640 (Affymetrix Inc., Santa Clara, CA, USA) at 45 °C for 16 h. Gene chips were washed and stained using Affymetrix Fluidics Station 450 according to the GeneChip Expression Analysis Technical Manual. Microarrays were scanned with the Affymetrix GeneChip Scanner 7G, and signals were processed with a target value of 600 using GCOS (version 1.4, Affymetrix Inc., Santa Clara, CA, USA).

Our study of whole genome expression profiling of hippocampal genes from aged 18 month-old Tg2576 mice is without precedent, *i.e.*, to date, hippocampal whole genome microarray gene expression data of aged 18 month-old Tg2576 mice are not available in the NCBI GEO database (search terms: Tg2576 and hippocampus). Due to the low amount of hippocampal tissue, hippocampal RNA from four Tg2576 mice was pooled for one gene chip, and two gene chips are presented for each group. Such an approach is valid because the inter-individual variability of inbred mouse lines is negligible as was analyzed for the Tg2576 model with RNA from brain cortex [52]. Another study with AD mice, which used 12 month-old Tg2576 mice (GSE1556), *i.e.*, the age of our control group, was also performed in duplicates [53,54]. Moreover, transgenic expression of a disease-causing gene (*i.e.*, APP^Swe^) and identical (inbred) genetic background (B6) accounts for a well-defined time course of disease progression of Tg2576 mice.

Selection criteria for differentially expressed genes (*p* ≤ 0.01, just alpha, no false discovery correction, and a two-fold change requirement) were specifically validated for chemical (drug) treatment effects and follow the guidelines of the MicroArray Quality Control (MAQC) project for the identification of reproducible gene lists [55,56]. Probe sets with significant difference (*p* ≤ 0.01, and ≤ −2-fold or ≥+2-fold difference, with call present and/or signal intensity ≥100) between treated Tg2576 mice relative to age-matched untreated Tg2576 mice were used for GO classification. Gene ontology (GO) analyses of microarray data were performed with GCOS-processed data using GeneSpring GX software (Agilent Technologies Inc., Santa Clara, CA, USA). Results were supported by RMA-normalized data (not shown). Major conclusions based on microarray data were validated by immunohistological and immunoblotting techniques performed with different groups of mice. All microarray gene expression data are available at the NCBI GEO database (accession number GSE46871).

### 3.3. Biochemical Analyses

ACE activity measurement of homogenized hippocampal tissue was performed with a fluorogenic substrate (Abz-FRK(Dnp)-P) according to the specifications of the manufacturer (Biomol International, Enzo Life Sciences AG, Lausen, Switzerland) in the absence and presence of captopril as described [28]. Activities of β-secretase and γ-secretase of homogenized hippocampal tissue were measured with fluorogenic substrates for each secretase (Calbiochem) as detailed previously [45]. Hippocampal angiotensin II level was determined by immunoblot after Tricine-SDS-PAGE with antibodies specific for angiotensin II displaying minimal crossreactivity with angiotensin I (<1%), as described [57]. Hippocampal content of Epha4 was determined by direct binding assay with anti-Epha4 antibodies followed by detection with ^125^I-labeled secondary antibodies (1.0 μCi/point; final concentration 5 × 10^−8^ M) similarly as described [20]. Binding assays were performed in triplicates with crude homogenates (0.5 mg of protein/mL) prepared from dissected hippocampi of 12 and 18 month-old Tg2576 mice, respectively, treated for six months without or with captopril or enalapril as indicated.

### 3.4. Immunohistology, Immunofluorescence and Immunoblotting

For immunohistology, paraffin-embedded brain sections (8 μm, taken at 50 μm intervals, 10–15 sections/set) were prepared from the different groups of Tg2576 mice. Sections were deparaffinized followed by antigen retrieval as detailed previously [20,57]. Immunohistological detection of ACE, AT1, and Epha4 was performed with F(ab)_2_ fragments of affinity-purified polyclonal antibodies pre-absorbed to mouse tissue. Sections were stained for Aβ plaques with monoclonal antibodies cross-reacting with residues 1–12 of the Aβ peptide (clone BAM-10; Sigma-Aldrich, St. Louis, MO, USA). All sections were imaged with a Leica DMI6000 microscope equipped with a DFC420 camera. Plaque burden of the different study groups was analyzed by computerized quantitative image analysis of immuno-stained hippocampal areas [20]. Immunofluorescence and dihydroethidium (DHE) staining were performed with cryo-sections (8 μm) of post-fixed and frozen brains obtained from the different study groups of Tg2576 mice. Sections were imaged with a confocal laser microscope (Leica TCS SPE). For co-localization studies of AT1 (or ACE) with Aβ, affinity-purified rabbit anti-AT1 antibodies (or rabbit anti-ACE antibodies) and monoclonal mouse Aβ antibody were applied (dilution 1:4000), followed by secondary antibodies labeled with Alexa Fluor 488 and Alexa Fluor 546, respectively (dilution of 1:5000; Molecular Probes, Life Technologies Corp., Carlsbad, CA, USA). Immunoblot detection of hippocampal proteins was performed with the guanidine hydrochloride-extracted protein fraction (6.25 M guanidine hydrochloride in 50 mM Tris, pH 8.0), similarly as described [20,21]. The hippocampal content of the APP intracellular domain (AICD), an APP cleavage product generated by γ-secretase, was determined by immunoblot with anti-APP^Cter^ antibodies after separation of cytosolic hippocampal proteins by Tricine-SDS-PAGE. SDS-insoluble, formic acid (70%)-extractable Aβ^1-40^ and Aβ^1-42^ content of hippocampi was determined by ELISA after serial tissue extraction in the presence of protease inhibitors as described [20]. Hippocampal 3-nitrotyrosine content was quantified by slot blot with 3-nitrotyrosine-specific antibodies (Sigma) similarly as described [58].

### 3.5. Antibodies

The following antibodies were used for immunohistology, immunofluorescence and immunoblotting: anti-ACE antibodies (raised in rabbit against an antigen corresponding to amino acids 720–750 of mouse ACE), anti-AT1 antibodies (raised in rabbit against an antigen corresponding to amino acids 306–359 of the mouse Agtr1a sequence), anti-Epha4 antibodies (raised in rabbit against an antigen corresponding to amino acids 917–927 of mouse Epha4), anti-Gnag2 antibodies (raised in rabbit against recombinant Gnag2), anti-Gnaq/11 antibodies (raised in rabbit against the *C*-terminus of Gnaq; [20]), anti-Kcnc1 antibodies (raised in rabbit against an antigen corresponding to amino acids 567–585 of mouse Kcnc1), anti-Klotho antibodies (raised in rabbit against an antigen corresponding to amino acids 998–1014 of mouse Klotho), anti-Rab6b antibodies (raised in rat against recombinant mouse Rab6b fusion protein), anti-Scn1a antibodies (raised in rabbit against an antigen corresponding to amino acids 465–481 of mouse Scn1a), anti-Transgelin antibodies (raised in rabbit against residues 16–90 of mouse Transgelin). Aβ plaques were stained with monoclonal anti-Aβ antibody (monoclonal antibody cross-reacting with residues 1–12 of the Aβ peptide; clone BAM-10, Sigma), sAPPβ (sw) was detected with a monoclonal antibody (6A1) specifically recognizing the β-secretase-cleaved end of APP^Swe^, and immunoblot detection of AICD was performed with anti-APP^Cter^ antibodies raised in rabbit against an antigen corresponding to residues 676–695 of APP695. Antibody specificity and cross-reactivity with the respective protein were routinely controlled by ELISA, immunoblotting and immunofluorescence applying the antigen used for immunization and cell lysates, or cells over-expressing the respective target gene.

### 3.6. Statistical Analysis

Unpaired two-tailed Student’s *t*-test was used to calculate *p* values. For comparisons between more than two groups, analysis of variance was performed with Prism (GraphPad) or TIGR MeV, followed by a Post-test as indicated. Statistical significance was set at a *p* value of <0.05 unless indicated otherwise.

## 4. Conclusions

Aβ plaque accumulation in the hippocampus of AD mice was retarded by treatment with the centrally active ACE inhibitor, captopril. Concomitantly, captopril promoted signs of neuronal regeneration as evidenced by gene expression profiling and immunohistological analyses. Treatment effects of captopril were accompanied by normalization of the AD-related increase in ACE protein level and activity, decreased accumulation of hippocampal angiotensin II and reduced ROS generation. In addition, the amyloidogenic processing of APP by β- and γ-secretase was decreased in captopril-treated AD mice. Together with previous studies [43–45], our data suggest a vicious cycle of Aβ-dependent ACE induction, ACE-dependent angiotensin II release, angiotensin II AT1 receptor activation-dependent ROS formation, which in turn promotes Aβ generation by enhancing the amyloidogenic processing of APP [7,45,47]. The thereby stimulated release of Aβ is expected to further up-regulate ACE [43,44] and to trigger a new round of angiotensin II-mediated Aβ generation (Figure 10).

Clinical evidence indicates beneficial effects of long-term antihypertensive treatment with a centrally active ACE inhibitor regarding retardation of the onset of cognitive decline [34]. The present study complements those data and suggests a synergism between beneficial vascular effects and inhibition of angiotensin II-promoted APP processing—at least in individuals with enhanced angiotensin II generation. Since the ACE protein level is also increased in the brains of patients with Alzheimer’s disease, and all components of the angiotensin-AT1 receptor system are present in different regions of the human brain [27,28,43], future studies will have to determine whether the neuro-regenerative potential of captopril documented in AD mice could be also of relevance for individuals at risk of developing cognitive impairment or AD.

## Supplementary Files

Supplementary File 1 Supplementary1 (XLS, 2289 KB)

Supplementary File 2 Supplementary2 (XLS, 2160 KB)

Supplementary File 3 Supplementary3 (XLS, 2182 KB)

Supplementary File 4 Supplementary4 (XLS, 74 KB)

Supplementary File 5 Supplementary5 (XLS, 47 KB)
